# Hahnemann’s *Organon*—A Query

**Published:** 1888-07

**Authors:** S. Lilienthal


### HAHNEMANN'S "ORGANON:" A QUERY.

DEAR EDITOR:—In my lecture on the *Organon* to-day I came to § 32, and I am sorry to say that I cannot fully agree with the Master, and beg, therefore, for an explanation. It reads:

"For every true medicine (drug) acts at all times, and under *all* circumstances, upon *every* living human being, and excites its peculiar symptoms in the organism (even very perceptibly if the dose is large enough). Thus every living human organism is always (*unconditionally*) affected, and, as it were, infected by the drug disease, which, as stated, is not at all the case with natural diseases."

The italics I find in Wesselhœft's edition, and my experience runs counter to that of the Master. I know a physician who took ten drops of a tincture several times, and, though a close observer, never felt the least symptom from even such a perceptible dose. Some of my students proving for me were bullet-proof to drug action in health, whether given high or low, but responded promptly to the simillimum when sick. In some provers the low potencies failed, but they responded to higher potencies and *vice versa*. Even the dictum of a Hahnemann may be taken *cum grano salis* without showing irreverence to this great man. I will be glad if you show me where *my* error lies.

Fraternally yours,

S. LILIENTHAL.

